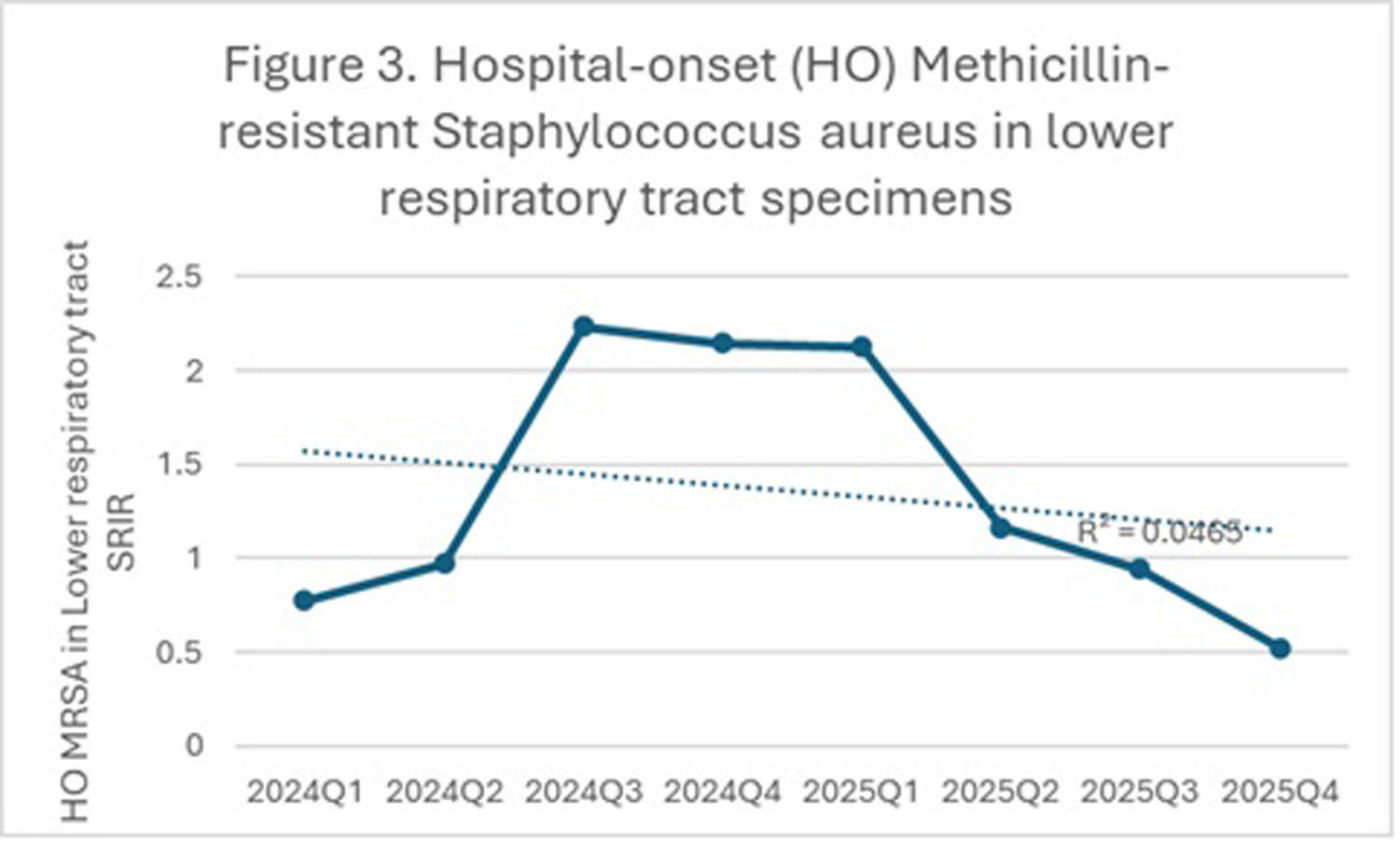# 175 Perceived Vulnerability to Respiratory Viral Infections Among Community Living Center Residents

**DOI:** 10.1017/ash.2026.10575

**Published:** 2026-06-23

**Authors:** Jennifer Gutowski, Emil Lesho, Shari McDonald, Melissa Bronstein, Maryrose Laguio-Vila, David Pierce

**Affiliations:** 1 Rochester Regional Health; 2 RGH

## Abstract

**Background:** Up to 33% of methicillin-resistant Staphylococcus aureus (MRSA) colonized patients will subsequently develop an invasive MRSA infection. Intranasal treatment is necessary to eliminate MRSA in the nose, which is recognized as a primary carriage site. In our 542 bed, community, teaching hospital, we had implemented CHG daily bathing for targeted patient populations (patients in ICU and with central lines), but struggled to reduce MRSA bacteremia rates. We proposed adding nasal decolonization and expanding the target population to include MRSA-positive patients, as a method of reducing MRSA bloodstream infections. **Methods:** On July 21, 2025, a formal MRSA decolonization protocol, including twice daily mupirocin for five days, and daily CHG bathing was recommended for all ICU patients, regardless of MRSA history, and MRSA positive patients, on any inpatient unit. A MRSA decolonization order set went live on October 27th, 2025, and was automatically added to pre-existing ICU admission and central line maintenance order sets. Pre-and post-intervention National Healthcare Safety Network (NHSN) hospital-onset MRSA bacteremia standardized infection ratios (SIR) were compared, in addition to a monthly review of MRSA bacteremia rates per 1000 patient days and mupirocin administration. NHSN antimicrobial resistance module standardized resistant infection ratios (SRIRs), were also compared between 2024 and 2025. **Result:** The pre-intervention (January-June 2025) MRSA bacteremia SIR was 0.853, compared to a post-intervention (August-December 2025) MRSA bacteremia SIR of 0.405. This represents a 53% reduction in SIR (p=0.398). The outpatient MRSA BSI prevalence rate from emergency department visits stayed steady between the pre and post-intervention periods, at 0.095 and 0.094. Figure 1 shows the inverse relationship between the increase in mupirocin administration (utilizing a threshold of < 5 administered doses) and decrease in hospital-onset MRSA bacteremia. Figures 2 and 3 show the prior years’ variation and current decline in SRIRs for both MRSA bacteremia and lower respiratory tract specimens. A decreasing trendline was not observed for other organisms’ SRIRs, including vancomycin-resistant Enterococcus. **Conclusion:** MRSA bacteremia contributes to significant morbidity, mortality, and financial penalties for hospitals. CHG bathing alone for ICU and central line patients was inadequate to reduce MRSA bacteremia rates. A default order set for MRSA decolonization, including nasal mupirocin, added to all ICU admissions and central line maintenance orders, improved compliance with the recommended protocol. Our hospital experienced a reduction in MRSA bacteremia following the implementation of a standardized decolonization protocol, including the nares, for all ICU, central line, and MRSA positive patients.

**Figure:**
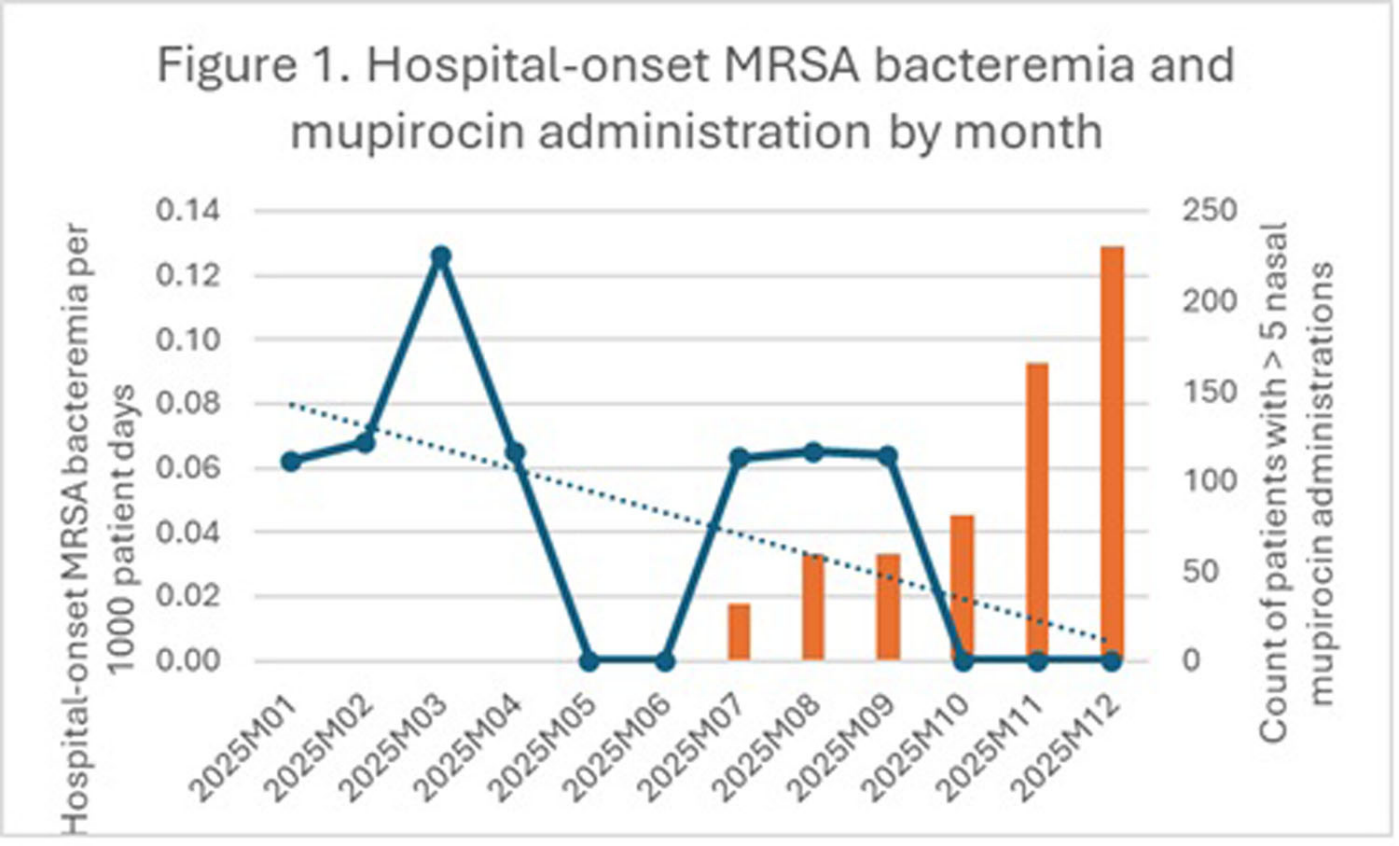

**Figure:**
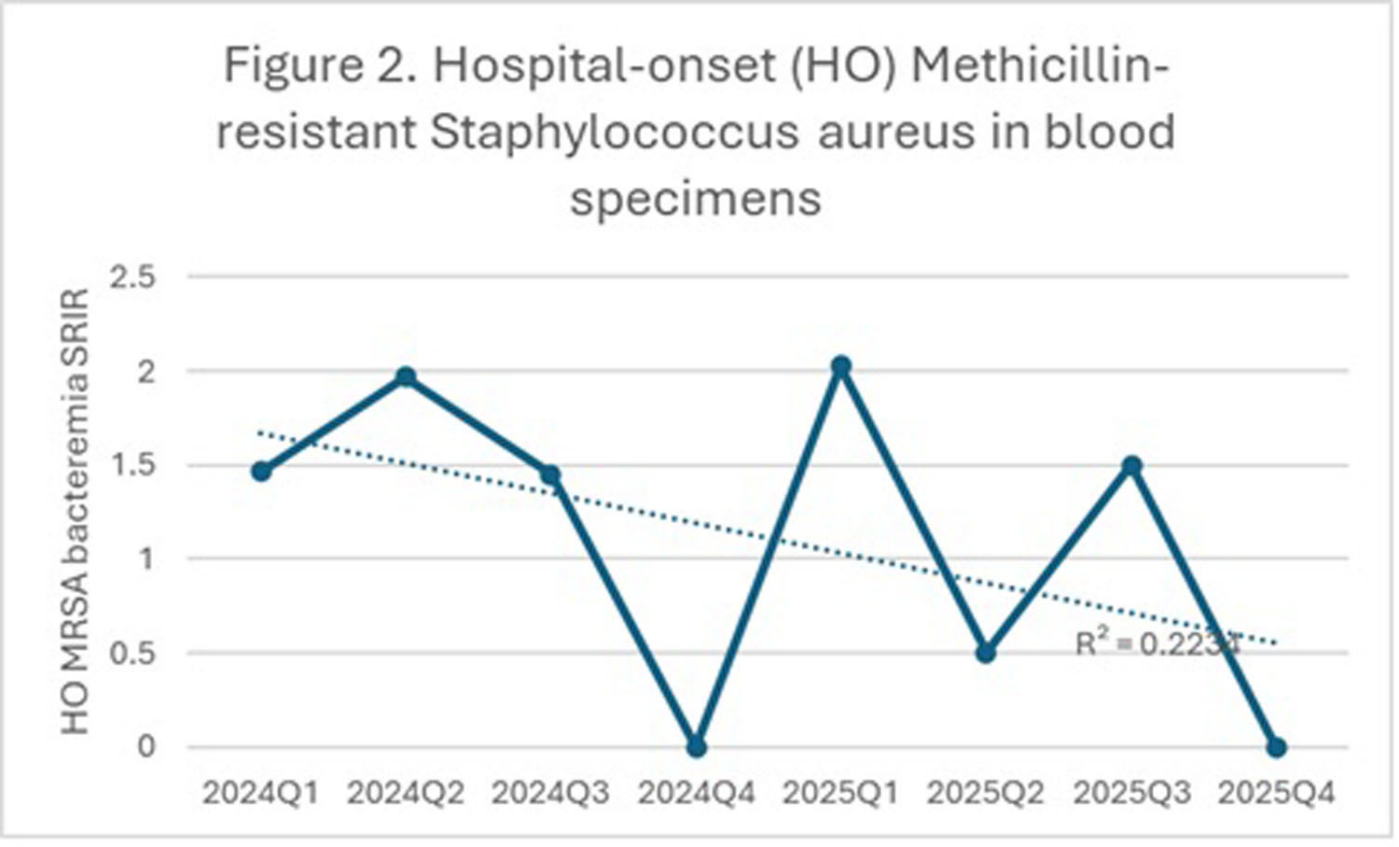

**Figure:**